# Opposite effect of ablation on early/late‐phase thromboembolic incidence in patients with atrial fibrillation: A meta‐analysis on more than 100 000 individuals

**DOI:** 10.1002/clc.23354

**Published:** 2020-03-11

**Authors:** Menghui Liu, Yuanping Wang, Jie Li, Xiaodong Zhuang, Xiaohong Chen, Xiaohui Li, Xinxue Liao, Lichun Wang

**Affiliations:** ^1^ Department of Cardiology The First Affiliated Hospital, Sun Yet‐sen University Guangzhou China; ^2^ Key Laboratory on Assisted Circulation Ministry of Health Guangzhou China; ^3^ The Second Affiliated Hospital Guangzhou University of Chinese Medicine Guangzhou China; ^4^ The Third Affiliated Hospital Sun Yet‐sen University Guangzhou China

**Keywords:** atrial fibrillation, catheter ablation, meta‐analysis, thromboembolism

## Abstract

**Background:**

Atrial fibrillation (AF) is an important risk factor for thromboembolic events, for which catheter ablation represents an effective therapy for rhythm control. Intuitively, ablation may reduce the incidence of thromboembolism, but data is quite limited.

**Hypothesis:**

Catheter ablation was associated with the fewer risk of thromboembolism compared with nonablation in patients with AF.

**Methods:**

A systematic search was performed in PubMed, EMBASE, the Web of Science, and the Cochrane Library from inception to September 2019. Random‐effects model was used to estimate the risk ratios (RR) for the thromboembolic events between the ablation and nonablation groups.

**Results:**

Twenty‐five studies (12 randomized controlled trials and 13 observational studies) with 104 687 participants were included. Pooled analysis suggested that ablation was associated with a 35% lower risk of total thromboembolic events compared to nonablation group (RR = 0.65; 95% CI, 0.51‐0.82; *P* = .0003). When separated into early‐phase (<30 days) and late‐phase (>30 days) events, ablation was associated with an increased early‐phase thromboembolism (RR = 1.96; 95% CI, 1.35‐2.83; *P* = .0004) but a decreased late‐phase thromboembolism (RR = 0.75; 95% CI, 0.63‐0.90; *P* = .002). Subgroup analysis according to different study types found similar results were found in observation studies, but not in RCT studies because the sample size was too small to be conclusive.

**Conclusions:**

In patients with AF, catheter ablation was associated with a fewer risk of overall and late‐phase thromboembolism in comparison with nonablation. However, over the early postoperative period, catheter ablation was associated with the double higher risk of thromboembolic events.

## 1 INTRODUCTION

Atrial fibrillation (AF), the most common form of cardiac arrhythmia, is an important risk factor for thromboembolic events, especially for ischemic stroke.^1^, ^2^ Thrombosis formation in patients with AF is mainly associated with slow blood flow and stasis of the left atrial appendage secondary to the loss of atrial rhythmic mechanical contraction.^3^ Based on this mechanism of thrombosis, effective rhythm control may reduce the incidence of thromboembolic events.

Catheter ablation, an effective method to restore and maintain sinus rhythm in patients with nonvalvular AF (NVAF),^4^ might reduce thromboembolic events following effective rhythm control. Theoretically, elimination of AF would abolish thrombogenesis in the left atrial appendage, and several observational studies have shown a relatively lower stroke rate after catheter ablation.^5^, ^6^, ^7^, ^8^, ^9^, ^10^, ^11^ Nevertheless, the current largest randomized controlled trials (RCTs) just showed a slight trend favoring the ablation in stroke events.^12^ Therefore, there is still not enough evidence to prove whether catheter ablation can reduce the thromboembolic risk until now. This study aimed to determine the effects of catheter ablation on thromboembolism and its possible characteristics in NVAF patients.

## 2 METHODS

### 2.1 Literature search

The protocol for this meta‐analysis was registered on PROSPERO with identifier CRD 42017056636 and published in the journal of Medicine (Baltimore).^13^ A systematic search was performed in PubMed, EMBASE, the Web of Science, and the Cochrane Library databases using the keywords “atrial fibrillation”, “ablation” and so on. The detailed search strategy of PubMed is in Table S1. The study population was humans, the published language was restricted to English, and all the studies were completed and published from inception to September 2019. The inclusion criteria included the following. (a) All of the recruited patients were ≥18 years and diagnosed with NVAF. (b) Patients in the experimental group received catheter ablation, while the control group was treated with nonablation therapy, including rhythm control with antiarrhythmic drugs, and rate control with or without antiarrhythmic drugs. (c) Study results reported thromboembolic events, including stroke, transient ischemic attack (TIA), and systemic embolic events. (d) Follow‐up of the studies was >6 months. Case reports, review articles, editorials, and duplicate reports were excluded.

### 2.2 Data extraction and quality assessment

Data from each study were extracted by two independent reviewers in accordance with the steps outlined in a predesigned schematic. Any disagreement on data abstracting was resolved by group discussion or arbitrated by a third author to reach consensus. The extracted information contained the design of the study, the baseline characteristics of the patients, the incidence of thromboembolic events, multivariable adjusted hazard ratio (HR) with 95% confidence intervals (CIs), anticoagulant strategy and follow‐up time. Among them, thromboembolic events included stroke, TIA, and systemic embolic events. Thromboembolic events were classified as early‐phase (which occurred within 30 days after ablation [ablation group] or enrollment [nonablation group]), late‐phase (>30 days after ablation [ablation group] or enrollment [nonablation group]) and total thromboembolic events according to the onset time. In the case of the studies including the same study cohort, only the most comprehensive or latest publication was eligible. The methodological quality and the risk of bias were also independently assessed by two reviewers. In RCTs, the risk of bias was assessed using the Cochrane Risk of Bias assessment tool from perspectives of selection bias, performance bias, detection bias, attrition bias, reporting bias, and other sources of bias.^14^ However, a modified version of the Newcastle‐Ottawa scale, which is a quality assessment tool for nonrandomized studies, was applied to appraise the quality of cohort studies or case‐control studies in three domains: the selection of participants, comparability of study groups, and the outcome of interest.^15^

### 2.3 Statistical Analysis

These data were analyzed using Review Manager 5.3 (The Cochrane Collaboration, Oxford, England), Stata (version 16.0, StataCorp, College Station, Texas), and trial sequential analysis (TSA; version 9.0, Copenhagen trial unit, Denmark). The results are presented as the rate ratio (RR) with 95% CIs and *P* values. In consideration of the possible heterogeneity among studies with regard to study types, study populations, anticoagulation strategy, timing, and primary endpoint, we only used random‐effects model to estimate the pooled effects. Moreover, the TSA were also performed with using TSA boundary to assess whether firm evidence was reached in cumulative meta‐analysis.^16^ The possible causes of clinical or methodological heterogeneity were explored by subgroup analysis or sensitivity analysis. In addition, in order to avoid the possible bias, adjusted estimates of effects were further performed in the pooled analysis of the observational studies. When sensitivity analysis was required, we removed each study to evaluate its effect on the remaining meta‐analysis. In accordance with Cochrane, evidence of publication bias was examined through funnel plots and Egger's test provided that there were more than 10 available studies.^17^

## 3 RESULTS

### 3.1 Search results

**Figure 1:**
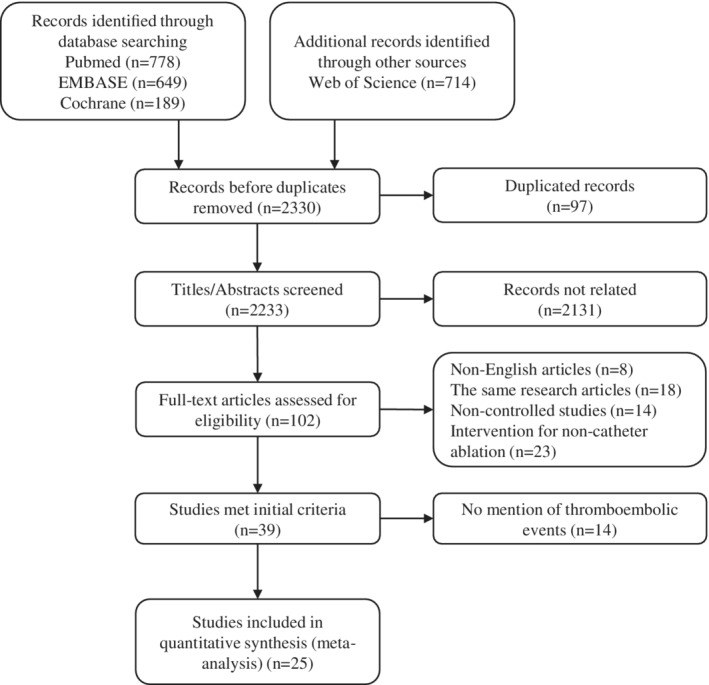
PRISMA flow diagram of selection in the study

A total of 2330 articles were initially retrieved from PubMed, EMBASE, the Web of Science, and the Cochrane Library. After removing duplicated and unrelated articles, 102 full‐text articles were assessed. Finally, 25 articles met the inclusion criteria and were included in this meta‐analysis (Figure 1). Twelve studies were RCTs^12^, ^18^, ^19^, ^20^, ^21^, ^22^, ^23^, ^24^, ^25^, ^26^, ^27^, ^28^ and the other thirteen studies were observational studies (ten retrospective studies^8^, ^9^, ^10^, ^11^, ^29^, ^30^, ^31^, ^32^, ^33^, ^34^ and three prospective cohort studies^6^, ^35^, ^36^).

### 3.2 Study characteristics

The study characteristics are shown in Table 1. A total of 104 687 patients (4082 in RCTs and 100 605 in observational studies) were involved in the 25 studies, of which the total number of thromboembolic events was 3602 (104 in RCTs and 3498 in observational studies). The average follow‐up time ranged from 6 to 144 months, and only three studies had a follow‐up <12 months. Fifteen studies (four RCTs and eleven observational studies) described the CHADS_2_ /CHA_2_DS_2_‐VASc scores and showed balanced scores between ablation and nonablation groups. Five of 13 observational studies revealed the adjusted HR in their articles. Additionally, the left atrial diameter, left ventricular ejection fraction, previous medical history, and the anticoagulant strategies were inadequately reported as shown in Table S2.

**Table 1:** Characteristics of the included studies^a^

			Age		Male (%)						CHADS_2_ score/CHA_2_DS_2_‐VASc score		
Study, year	Type of study	No. of patients, n	Ablation	Nonablation	Ablation	Nonablation	Average follow‐up (months)	Experimental group	Control group	Types of AF	Ablation	Nonablation	Multivariable adjustment HR (95%Cl)
Raatikainen, 2015	RCT	286	56 ± 10	56 ± 10	70.62	68.47	24	RFA or crossover	AADs	PAF	0.47 ± 0.80/NA	0.66 ± 0.76/NA	—
Mont, 2014	RCT	146	55 ± 9	55 ± 9	77.5	77.0	12	RFA	AADs	Persistent AF	NA	NA	—
Morillo, 2014	RCT	127	56.3 ± 9.3	56.3 ± 9.3	77.27	73.77	24	RFA	AADs	PAF	0.5 ± 0.7/NA	0.7 ± 0.8/NA	—
Pappone, 2011	RCT	198	55 ± 10	57 ± 10	69.70	64.65	12	RFA	AADs	PAF	NA	NA	—
Wilber, 2010	RCT	167	55.5	56.1	68.9	62	9	RFA	AADs	PAF	NA	NA	—
Jais, 2008	RCT	112	49.7 ± 10.7	52.4 ± 11.4	84.9	83.1	12	RFA	AADs	PAF	NA	NA	—
Oral, 2006	RCT	146	55 ± 9	58 ± 8	87	90	12	RFA	Amiodarone	Chronic AF	NA	NA	—
Bertaglia, 2017	RCT	137	62.2 + 8.9	62.3 + 10.7	54.4	63.8	144	RFA	AADs	PAF (67.15%)	NA	NA	—
Wazni, 2005	RCT	70	53 ± 8	54 ± 8	NA	NA	12	RFA	AADs	PAF (95.71%)	NA	NA	—
Hummel, 2014	RCT	210	59.6 ± 8.3	60.7 ± 8.9	83.3	83.3	6	RFA	AADs	Persistent AF (72.86%)	0.8 ± 0.8/NA	0.8 ± 0.7/NA	—
Marrouche, 2018	RCT	363	56‐71	56‐73.5	87	84	37.8 (37.6 ± 20.4)	RFA	AADs	PAF (32.51%)	NA	NA	—
Packer, 2019	RCT	2204	68 (62‐72)	67 (62–72)	62.7	63	48.5 (29.9‐62.1)	RFA	AADs	PAF (43.00%)	NA/3.0 (2.0, 4.0)	NA/3.0 (2.0, 4.0)	—
Blandino, 2013	Prospective cohort study	412	75 ± 5	76 ± 5	71	72	60 ± 17	RFA	AADs	Persistent AF	NA	NA	NA
Bai, 2015	Prospective cohort study	222	61.82 ± 8.90	62.42 ± 10.52	63.51	62.84	6	RFA	Nonablation	PAF (60.00%)	0.62 ± 0.49/NA	0.64 ± 0.48/NA	NA
Bunch, 2013	Prospective cohort study	21 060	64.8 ± 12.7	66.0 ± 13.3	60.8	60.8	12	RFA	Nonablation	NA	1.26 ± 1.33/NA	1.33 ± 1.37/NA	NA
Gallo, 2016	Retrospective cohort study	1500	61 ± 9	70 ± 9	68	57.4	60 ± 28	RFA	Rate control	PAF (33.87%)	NA/2.1 ± 1.1	NA/3 ± 1.3	NA
Noseworthy, 2015	Retrospective cohort study	24 244	>50(81.6%)	>50(81.7%)	74.15	74.90	28.8 ± 21.6	RFA	Cardioversion	NA	0–1:7326, >2:4796	0–1:7309, >2:4813	NA
Lin, 2012	Retrospective cohort study	348	57 ± 10	57 ± 11	52.9	53.4	47 ± 23	RFA	AADs	PAF (73.28%)	1.10 ± 0.84/NA	1.15 ± 1.00/NA	NA
Reynolds, 2012	Retrospective cohort study	1602	>50(90.8%)	>50(90.6%)	60.92	62.55	36	RFA	Nonablation	NA	0.98 ± 0.97/NA	1.00 ± 0.97/NA	0.60(0.42, 0.84)
Chang, 2014	Retrospective cohort study	12 170	51.91 ± 15.30	66.98 ± 12.69	70.8	59.33	42	RFA	Nonablation	NA	0.56 ± 0.73/NA	1.08 ± 0.85/NA	0.57(0.39, 0.94)
Friberg, 2016	Retrospective cohort study	4992	59.97 ± 10.20	59.55 ± 12.83	75.8	76.2	52.8 ± 24	RFA	Nonablation	NA	NA/1.62 ± 1.44	NA/1.62 ± 1.44	0.69(0.51, 0.93)
Jarman, 2017	Retrospective cohort study	20 796	58.79 ± 10.72	58.8 ± 10.75	69.75	69.65	60	RFA	Nonablation or Cardioversion	NA	0.49 ± 0.68/ 1.23 ± 1.21	0.48 ± 0.68/ 1.22 ± 1.18	NA
Saliba, 2017	Retrospective cohort study	4741	69.36 ± 4.07	69.37 ± 4.04	63.3	63.7	36	RFA	Nonablation	NA	1.9 ± 1.4/ 3.6 ± 2.0	1.9 ± 1.4/ 3.6 ± 2.0	0.58(0.43, 0.72)
Srivatsa, 2018	Retrospective cohort study	8338	>50(84.6%)	>50(85.8%)	72.3	71.2	43.2 ± 10.8	RFA	Nonablation	NA	NA	NA	0.76(0.54, 1.10)
Geng, 2017	Retrospective cohort study	394	64.7 ± 9.4	65.4 ± 11.4	50.0	45.6	13.5 ± 5.3	RFA	Rate control	NA	NA/2.3 ± 1.5	NA/2.5 ± 1.3	NA

Abbreviations: AADs, antiarrhythmic drugs; AF, atrial fibrillation; CI, confidence interval; HR, hazard ratio; NA, not available; RCT, randomized, controlled trial; RFA, radiofrequency ablation; PAF, paroxysmal atrial fibrillation.

a Plus‐minus values are means ± SD and medians (25‐75 percentiles) present non‐normally distributed data.

### 3.3 Risk of bias assessment

Assessment of the risk of bias for the 12 RCTs is shown in Figure S1. Outcomes were blindly assessed in five RCTs, and outcomes of the remaining RCTs were evaluated by the referee. For the 13 observational studies, the risk of bias was assessed using the Newcastle‐Ottawa Scale (Table S3), resulting in 8/9 points in four studies, 7/9 in five studies, 6/9 in three studies, and 5/9 in one study. Evidence of publication bias was assessed using a funnel plot and Egger's tests. In RCTs, the funnel plot indicated publication bias might exist (Figure. S2), and further Egger's tests showed the publication bias had a statistical trend (*P* = .060). The funnel plot of observational studies was almost symmetrical (Figure. S2) and Egger's tests showed no statistical difference (*P* = .826).

### 3.4 Total thromboembolic event analysis

**Figure 2:**
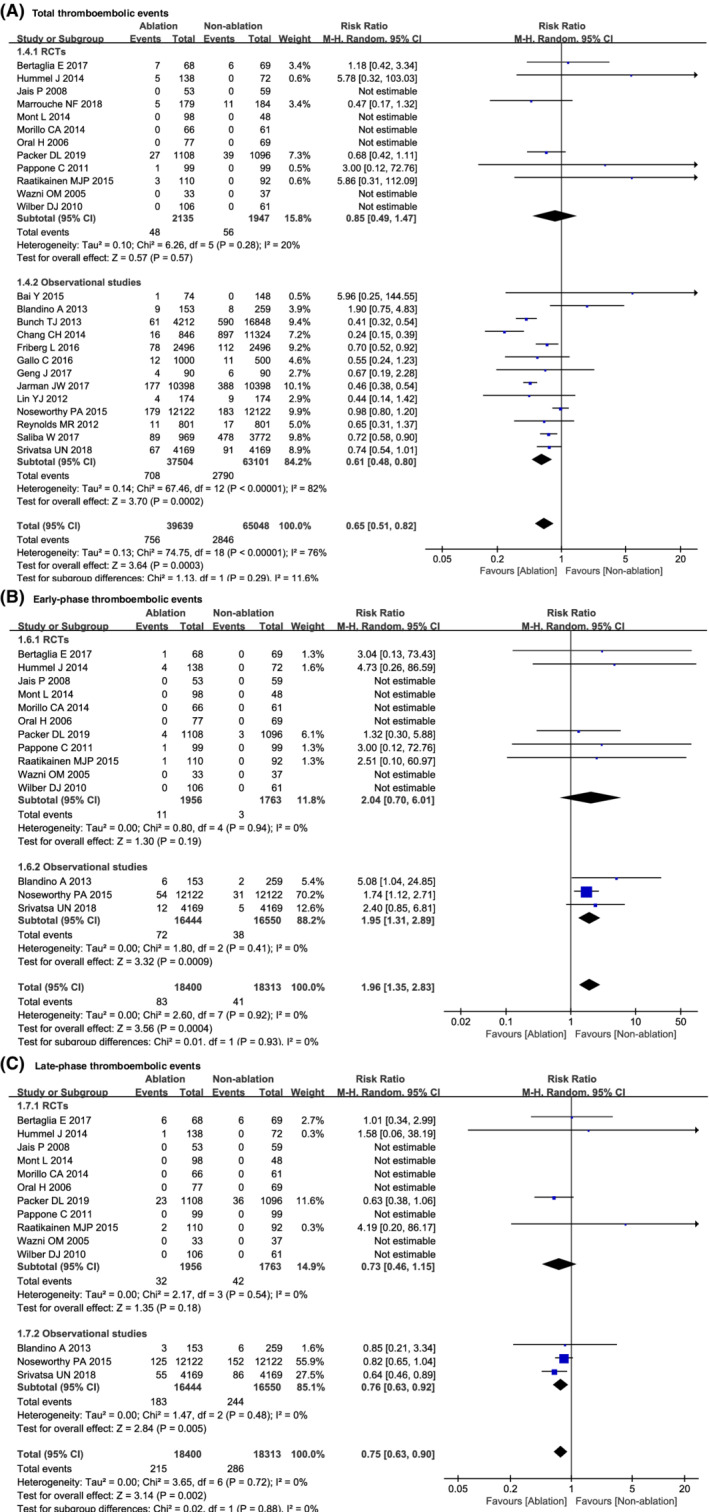
Comparison of the incidence of thromboembolism between ablation and nonablation

Pooled analysis among the 25 studies showed that the incidence of total thromboembolic events was 756 of 39 639 (1.91%) patients in the ablation group and 2846 of 65 048 (4.38%) patients in the nonablation control group. Catheter ablation was associated with a 36% lower risk of total thromboembolic events compared to nonablation control group (RR = 0.65; 95% CI, 0.51‐0.82; *P* = .0003; *I* ^2^ = 76%; Figure 2A).

The subgroup analysis was further performed according to different study types. In subgroups of 13 observational studies, there was also significant difference in total thromboembolic events between the ablation group and the control group (RR = 0.61; 95% CI, 0.48‐0.80; *P* = .0002; *I* ^2^ = 82%). However, the differences were not found in 12 RCTs (RR = 0.85; 95% CI, 0.49‐1.47; *P* = .57; *I* ^2^ = 20%; Figure 2A.

In addition, because only five studies described the adjusted HR in the 13 observational studies, pooled analysis was performed in these five studies further using adjusted estimates of effects. The result also exhibited that ablation was associated with a 36% lower risk in comparison with nonablation control group (HR = 0.64; 95% CI, 0.55‐0.74; *P* < .0001; *I* ^2^ = 0%; Figure S3).

To eliminate the bias caused by the possible difference of CHADS_2_/CHA_2_DS_2_‐VASc scores, subgroup analysis in the 15 studies (4 RCTs and 11 observational studies), which had balanced CHADS_2_/CHA_2_DS_2_‐VASc scores between groups, showed the total thromboembolic events was significantly reduced in the ablation group (RR = 0.60; 95%CI, 0.46‐0.78; *P* = .0001). Similar results were also found in the 11 observational studies (RR = 0.56; 95% CI, 0.43‐0.75; *P* < .0001; I^2^ = 83%). However, in the four RCTs pooled analysis did not show statistically difference (RR = 1.69; 95% CI, 0.32‐8.93; *P* = .53; *I* ^2^ = 51%; Figure S4).

### 3.5 Analysis of early‐phase thromboembolic events

Of the 25 included studies, 14 studies (11 RCTs and 3 cohort studies) described early‐phase and late‐phase thromboembolic events. Pooled analysis in these 14 studies showed the double higher risk of early‐phase thromboembolic events in the ablation group than in the nonablation group (RR = 1.96; 95% CI, 1.35‐2.83; *P* = .0004; *I* ^2^ = 0%; Figure 2B).

Subgroup analysis in RCTs indicated a slight trend favoring the nonablation group (RR = 2.04; 95% CI, 0.70‐6.01; *P* = .19; I^2^ = 0%). In observational studies, the incidence of early‐phase thromboembolic events was significantly increased in the ablation group (RR = 1.95; 95% CI, 1.31‐2.89; *P* = .0009; *I* ^2^ = 0%; Figure 2B).

Further subgroup analysis in five studies (four RCTs and one observational study) that described early‐phase thromboembolic events and balanced CHADS_2_/CHA_2_DS_2_‐VASc scores also showed the nonablation group was superior to the ablation group in early‐phase thromboembolic events (RR = 1.75; 95% CI, 1.16‐2.65; *P* = .008; *I* ^2^ = 0%). In fact, this result was majorly driven by the one observational study (85 events in 24 244 patients). In the four RCTs, only twelve thromboembolic events occurred, pooled analysis did not show significance between the two groups (RR = 1.85; 95% CI, 0.53‐6.21; *P* = .34; *I* ^2^ = 0%; Figure S4).

### 3.6 Analysis of late‐phase thromboembolic events

In the 14 studies (11 RCTs and 3 cohort studies) that reported early‐phase and late‐phase thromboembolic events, pooled analysis indicated the late‐phase thromboembolic events were significantly fewer in the ablation group (RR = 0.75; 95% CI, 0.63‐0.90; *P* = .002; *I* ^2^ = 0%; Figure 2C).

Subgroup analysis in observational studies also indicated the late‐phase thromboembolic events was significantly fewer in the ablation group (RR = 0.76; 95% CI, 0.63‐0.92; *P* = .005; *I* ^2^ = 0%). Additionally, there was a tendency favoring the ablation group compared to the control group in RCTs (RR = 0.73; 95% CI, 0.46‐1.15; *P* = .18; *I* ^2^ = 0%; Figure 2C).

In the five balanced CHADS_2_/CHA_2_DS_2_‐VASc scores studies (four RCTs and one observational study), further analysis showed catheter ablation was associated with a fewer risk of late‐phase thromboembolic events in comparison with nonablation (RR = 0.79; 95% CI, 0.64‐0.98; *P* = .03; *I* ^2^ = 0%), although no differences were found in the RCTs subgroup (RR = 0.68; 95% CI, 0.41‐1.13; *P* = .13; *I* ^2^ = 0%) and in the observational study subgroup (RR = 0.82; 95% CI, 0.65‐1.04; *P* = .10; *I* ^2^ = 0%; Figure S4).

### 3.7 Subgroup analysis of the long‐term follow‐up studies

Considering that the number of thromboembolic events was associated with the follow‐up length of included studies, we further analyzed the 22 long‐term follow‐up studies (follow‐up time ≥12 months). The results also showed that catheter ablation was associated with the fewer risk of total (RR = 0.63; 95% CI, 0.50‐0.80; *P* = .0001; *I* ^2^ = 77%) and late‐phase thromboembolism (RR = 0.75; 95% CI, 0.63‐0.90; *P* = .002; *I* ^2^ = 0%) in patients with AF, but with the higher risk of early‐phase thromboembolism (RR = 1.93; 95% CI, 1.33‐2.80; *P* = .0005; *I* ^2^ = 0%; Figure S5).

### 3.8 Sensitivity analysis

**Figure 3:**
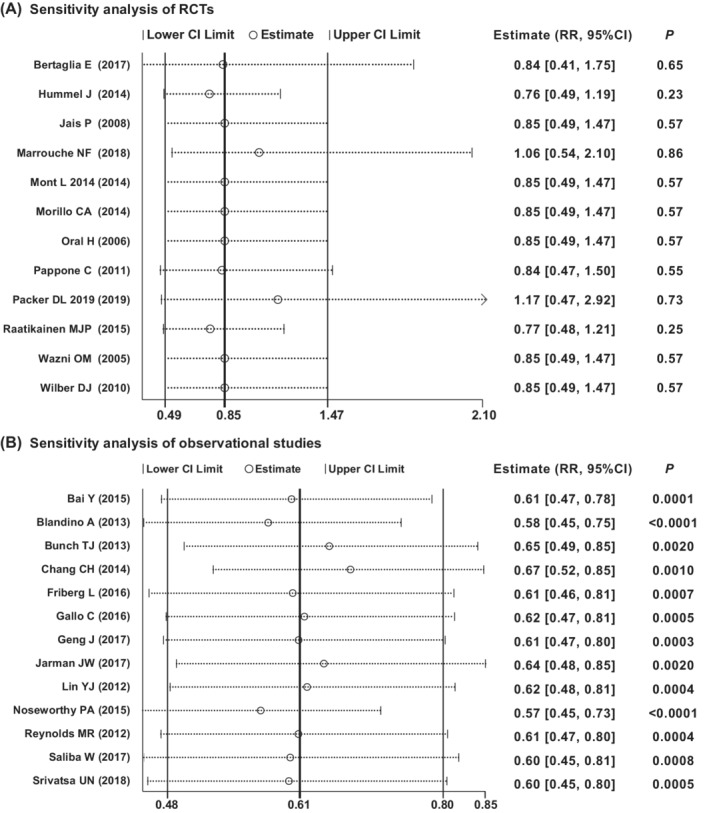
Sensitivity analysis of total thromboembolic events

Sensitivity analysis of the total thromboembolic events was respectively performed in RCTs and observational studies (Figure 3). After removing each study in RCTs, the pooled analysis results of the remaining (*P* = .23‐.86) were consistent with the previous meta‐analysis (*P* = .57). Similarly, the removal of each study in observational studies also did not change the result of pooled analysis. These results indicated single study had no significant effect on the results of pooled meta‐analysis.

In addition, since the accurate differential diagnosis of TIA was often difficult, we further perform a sensitivity analysis with only stroke and systemic embolism in the 11 studies that clearly distinguished the different types of thromboembolic events (TIA, stroke, and systemic embolic events). The similar results to the primary results were also found (Figure S6).

### 3.9 Reliability analysis by TSA

**Figure 4:**
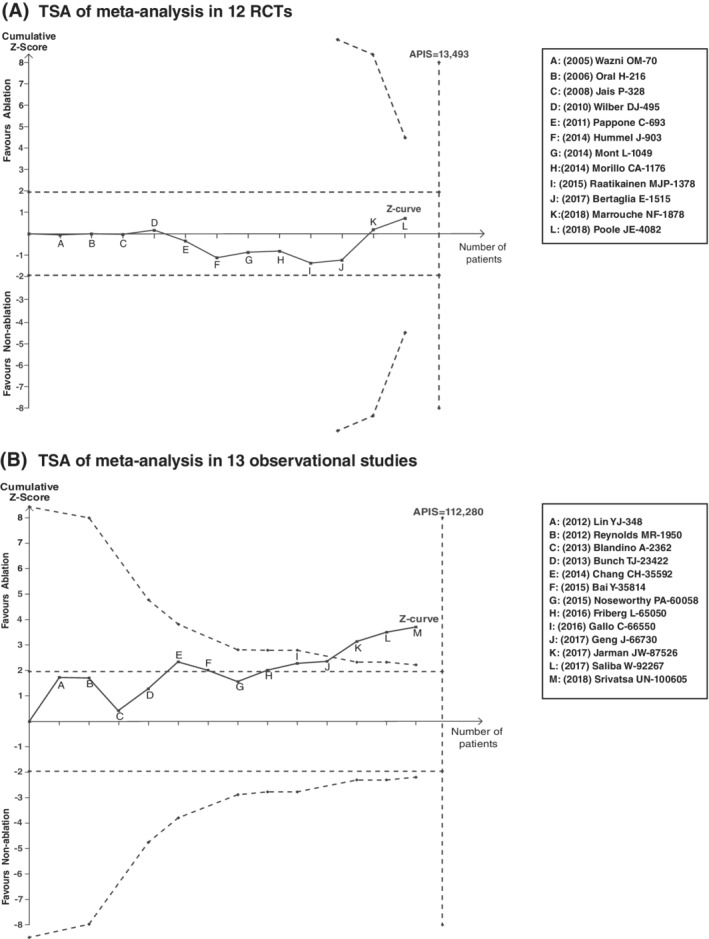
Trial sequential analysis (TSA) of meta‐analysis in 12 RCTs and 13 observational studies. APIS: information size calculated from an a priori assumed intervention effect

Because the incidence of ischemic stroke is ~5% per year in patients with AF,^2^ we set the 5% as control event rate to estimate the optimal sample size (APIS) with 20% relative risk reduction, 80% power and 0.05 two sided. The results indicated that the APIS were at least 13 493 patients in 12 RCTs and 112 280 patients in 13 observational studies. Unfortunately, only a total 4082 patients were involved in the whole RCTs and the Z‐curve line did not cross the TSA boundary (Figure 4A). This highly indicated that the pooled analysis only on RCTs was inconclusive. As for observational studies, however, the Z‐curve line obviously crossed the TSA boundary, although the involved patients (100605) were just a little less than the APIS (Figure 4B). Thus, the pooled results from observational studies were reliable and conclusive.

## 4 DISCUSSION

We performed a meta‐analysis to compare the incidence of thromboembolic events between NVAF patients with and without catheter ablation. And the results of all included studies showed that catheter ablation was associated with a 35% lower risk of overall thromboembolism and a 25% lower risk of late‐phase events compared to the nonablation group, but that was associated with the double higher risk of early‐phase thromboembolism.

However, in the subgroup analysis according to the study type, it was just in observational studies that catheter ablation could be found to have the above effects. In RCTs there was no significant difference between the ablation and control group, although a trend favoring nonablation control group in early‐phase thromboembolism and favoring the ablation group in late‐phase thromboembolism. There might be several reasons for this difference.

First of all, the relatively small sample size in RCTs might be the critical factor. Comparing to the observation studies (n = 100 605; events = 3498), the number of the involved patients (n = 4082) and occurred events (n = 104) were very smaller, so it might be difficult to detect the potential difference between the test groups. In fact, the results from TSA showed the sample size in RCTs was far from the optimal sample size (13 493 patients).

Moreover, the bias assessment with the funnel plot and Egger's test showed there might be some publication bias in RCTs. This might also affect the statistical results, as recommended by the Cochrane Collaboration.^37^ However, the publication bias was not found in observational studies, and the total incidence of thromboembolic events in the nonablation control group was 4.38% (2846/65048) in these 25 studies. This incidence rate was similar to previous reports,^2^ and further supported the rationality of the results. Another possible reason was that the antithrombotic therapy is supervised better in the RCTs, minimizing the difference in thrombotic risk between the groups. Thus, the statistical difference was hard to be found in the analysis of RCTs with limited sample size. Nevertheless, future larger RCTs to confirm this view are indispensable.

Considering that the risk of thromboembolic events was highly related with CHADS_2_/CHA_2_DS_2_‐VASc scores, the subgroup analysis was performed in the 15 studies (4 RCTs and 11 cohort studies), and the results also demonstrated significantly fewer total thromboembolic events in the ablation group than the nonablation group in the all 15 studies or in the 11 observational studies. Simultaneously, we also performed the pooled analysis using adjusted estimates of effects in the 5 observational studies which described the adjusted HR in their articles, the sensitivity analysis with only stroke and systemic embolism in 11 included studies, and the subgroup analysis in the 22 long‐term follow‐up studies (follow‐up time ≥ 12 months). These results further indicated that catheter ablation in AF was associated with a lower risk of thromboembolic events.

Interestingly, 14 studies (11 RCTs) reported early‐phase (less than 30 days after ablation) and late‐phase (more than 30 days after ablation) thromboembolic events. Pooled analysis showed the incidence of early‐phase thromboembolism was significantly higher in the ablation group than in the nonablation group, whereas the late‐phase thromboembolic events were just opposite in all 14 studies or in observational studies subgroup. As for the increased incidence of the early‐phase thromboembolism, the reason might be due to the use of catheters and sheaths^38^ and endothelial lesions of the vasculature and heart during the ablation procedure.^39^ Additionally, the weeks or months atrial myocardium stunning postprocedure might also be one of the causes for increased perioperative thromboembolic events in AF ablation.^40^ So the expert consensus statement on catheter and surgical ablation of AF in 2017 still recommended systemic anticoagulation was necessary at least 2 months post catheter ablation of AF.^41^ In fact, the practice of anticoagulation during the perioperative period of AF ablation is always a focus of research. Recent studies have showed that the incidence of thromboembolic events (0.15%‐0.25%) is significantly reduced under uninterrupted warfarin^42^ or novel oral anticoagulation therapy^43^ compared with temporary discontinuation of anticoagulation in the perioperative period of AF ablation. As a result, it is still necessary to optimize the regime of anticoagulation during perioperative period of AF ablation. As for late‐phase thromboembolism, the pooled analysis showed ablation was also associated with a lower risk in all 14 studies or in observational studies subgroup. According to these results, it might be considerable to re‐evaluate the anticoagulation regimen in the patients who kept in sinus rhythm after 3‐month postablation, although the current ESC and AHA/ACC/HRS guidelines still recommend that oral anticoagulation after catheter ablation should follow general anticoagulation recommendations regardless of the presumed rhythm.^44^, ^45^ Therefore, further studies, such as focusing on the diversity of different heart rhythm outcome, were needed.

There are limitations in this study. Most importantly, the anticoagulant strategy of AF had an important effect on thromboembolic events. And this might be the cause for the mass variability of thromboembolic events among different studies. For example, the CABANA study, which is the current largest RCT in this field, the incidences of thromboembolic events were 27/1108 in ablation group and 39/1096 in drug therapy group^12^; but in Bertaglia et al study, the events occurrences were 7/68 and 6/69 in ablation and control groups, respectively.^25^ Although it was believed that the ablation group and the control group should had the same anticoagulation regimen in RCTs owing to the principle of homogeneity, unmatched probability might be existed in observational studies. Therefore, the meta‐analysis of uncorrected anticoagulation intensity might be biased. However, there were 6 observational studies and 12 RCTs that described the anticoagulation strategies between the two groups. If further subgroup analysis was performed in these 6 observational studies, the results still indicated that the incidence of total thromboembolic events was markedly reduced in the ablation group (RR = 0.63; 95% CI, 0.43‐0.93; *P* = .02; *I* ^2^ = 71%). The similar results were also found in the 18 studies (RR = 0.69; 95% CI, 0.51‐0.94; *P* = 0.02; *I* ^2^ = 56%; Figure S7). Second, because of the absence of a standard method of catheter ablation for AF, especially for persistent AF, the methods used might have difference among studies, even among individual patients within the same study. Therefore, the diversity of the catheter ablation method was not considered in our analysis. This might have caused some bias in the results. The results thus should be interpreted prudently owing to these limitations.

## 5 CONCLUSIONS

These findings indicated that catheter ablation was associated with a 35% lower risk of overall thromboembolism similar to late‐phase events compared with nonablation in patients with AF. However, over the early postoperative period, catheter ablation was associated with double higher risk of thromboembolic events indicating the necessity of optimizing the anticoagulation regime during the perioperative period of AF.

## CONFLICT OF INTEREST

The authors declare no potential conflict of interests.

## Supporting information


**Appendix** S1. Supporting Information
