# Supplementary material for: Opposite effect of ablation on early/late‐phase thromboembolic incidence in patients with atrial fibrillation: A meta‐analysis on more than 100 000 individuals
Source: Clin Cardiol. 2020 Mar 11;43(6):594–605. doi: 10.1002/clc.23354 (PMC7298999; doi:10.1002/clc.23354)
Supplement: Supplementary file 1 — Appendix S1. Supporting Information [file CLC-43-594-s001.pdf]

Supplementary Data

Table S1. The detailed search strategy of PubMed.

# 1	atrial fibrillation
# 2	non-valvular atrial fibrillation
# 3	paroxysmal atrial fibrillation
# 4	persistent atrial fibrillation
# 5	permanent atrial fibrillation
# 6	lone atrial fibrillation
# 7	#1 OR #2 OR #3 OR #4 OR #5 OR #6
# 8	ablation
# 9	radiofrequency catheter ablation
#10	radiofrequency ablation
#11	catheter ablation
#12	#8 OR #9 OR #10 OR #11
#13	thromboembolic incidence
#14	thromboembolic event
#15	thromboembolism
#16	stroke
#17	transient ischemic attack
#18	systemic embolic event
#19	#13 OR #14 OR #15 OR #16 OR #17 OR #18
#20	randomized controlled trial
#21	observational study
#22	cohort study
#23	prospective cohort study
#24	retrospective cohort study
#25	#20 OR #21 OR #22 OR #23 OR #24
#26	#7 AND #12 AND #19 AND #25

Table S2. Other characteristics of the enrolled studies

Study, year	Hypertension (%)		Heart Failure (%)		Diabetes Mellitus (%)		Thromboembolic Disease (%)		Left atrial diameter (mm)		Left ventricle ejection fraction (%)		Anticoagulant strategy	
	ablation	non-ablation	ablation	non-ablation	ablation	non-ablation	ablation	non-ablation	ablation	non-ablation	ablation	non-ablation	ablation	non-ablation
Raatikainen MJP, 2015	27.84	43	NA	NA	4.64	5	3.61	3	40±5	40±5	63±10	64±7	According to guidelines	
Mont L, 2014	46.9	39.5	NA	NA	NA	NA	4.08	4.17	41.3±4.6	42.7±5.1	61.1±8.8	60.8±9.7	At least 30d after ablation*	According to guidelines
Morillo CA, 2014	42.4	41	3.0	1.6	1.5	6.6	4.6	6.6	40±5	43±5	61.4±4.8	60.8±7.0	At least 90d after ablation*	According to guidelines
Pappone C, 2011	56	57	NA	NA	5.1	4	NA	NA	40±6	38±6	60±8	61±6	Discontinuity of OAC if SR was maintained for >6 weeks	
Wilber DJ, 2010	48..6	50	NA	NA	9.5	12	3.8	8	40.0±1.1	40.5±1.5	62.3±2.0	62.7±2.0	At least 90d after ablation*	According to guidelines
Jais P, 2008	0	3.4	NA	NA	1.9	3.4	1.9	11.9	39.5±5.6	40.0±5.7	63.1±11.0	65.6±7.2	At least 30d after ablation*	Continuation of anticoagulation
Oral H, 2006	NA	NA	NA	NA	NA	NA	NA	NA	45±6	45±5	55±7	56±7	Discontinuity of OAC if SR was maintained for >6 months	
Bertaglia E, 2017	52.9	49.3	NA	NA	NA	NA	NA	NA	46±5	45.4±5.5	59.1±6.7	57.9±5.8	OAC or antiaggregant therapy	
Wazni OM, 2005	25	28	NA	NA	NA	NA	NA	NA	41±8	42±7	53±5	54±6	At least 90d after ablation*	Continuation of anticoagulation
Hummel J, 2014	60.9	55.6	5.8	11.1	15.9	11.1	NA	NA	45±5	46±5	54.7±7.1	54.9±6.7	Continuation of anticoagulation	

Marrouche NF, 2018	NA	NA	100	100	NA	NA	NA	NA	45.0-54.0	50.0-55.0	25.0-38.0	27.0-37.0	At least 180d after ablation*	According to guidelines
Packer DL, 2019	79.1	82.2	15.7	14.9	25.3	25.7	10.6	9.4	NA	NA	NA	NA	All patients received anticoagulation	
Blandino A, 2013	70	74	NA	NA	3	6	17	14	47±5	46±5	56±6	56±8	At least 90d after ablation*	According to guidelines
Bai Y, 2015	51.35	55.41	NA	NA	5.41	3.38	NA	NA	39.09± 6.94	38.84± 6.23	64.21± 9.66	63.63± 9.31	NA	NA
Bunch TJ, 2013	47.8	45.3	45.3	29.5	16.3	21.1	NA	NA	NA	NA	51.3±13.8	56.1±15.9	Determined by electrophysiologist	
Gallo C, 2016	75	78	5	13.2	8.5	18	6.4	9.2	45±6	49±7	NA	NA	At least 90d after ablation*	Continuation of anticoagulation
Noseworthy PA, 2015	NA	NA	NA	NA	NA	NA	NA	NA	NA	NA	NA	NA	NA	NA
Lin YJ, 2012	70.1	64.9	13.2	16.7	17.2	17.8	2.9	4.6	39.4±6.84	40.1±9.00	57.5±8.89	57.0±9.63	At least 90d after ablation*	Continuation of anticoagulation
Reynolds MR, 2012	42.7	40.7	17.35	15.73	18.73	15.23	2.62	4.87	NA	NA	NA	NA	Continuation of anticoagulation	
Chang CH, 2014	38.18	59.51	0	0	10.87	17.41	0	0	NA	NA	NA	NA	NA	NA
Friberg L, 2016	47.5	48.6	15.2	16.0	10.0	10.3	14.6	14.4	NA	NA	NA	NA	NA	NA
Jarman JW, 2017	NA	NA	NA	NA	NA	NA	NA	NA	NA	NA	NA	NA	NA	NA
Saliba W, 2017	71.8	72.1	28.7	27.7	32.9	32.9	16.9	15.6	NA	NA	NA	NA	Anticoagulant patients (51.8%)	Anticoagulant patients (62.6%)

Srivatsa UN, 2018	58.1	51.1	11.6	12.7	14.1	20.4	4.7	3.8	NA	NA	NA	NA	NA	NA
Geng J, 2017	54.4	55.6	100	100	20.0	13.3	13.3	12.2	4.9±0.7	5.0±0.7	41.9±6.9	41.4±7.0	NA	NA

NA: not available; OAC: oral anticoagulation; SR: sinus rhythm. *Subsequent use of anticoagulation during the effectiveness evaluation period followed current guidelines.

Table S3. Results of quality assessment for cohort studies by the Newcastle–Ottawa Scale

study	1	2	3	4	5A	5B	6	7	8	score
Blandino A, 2013	★	★	★	★	−	★	−	★	−	6
Bai Y, 2015	★	★	−	★	★	★	−	★	★	7
Gallo C, 2016	★	−	★	★	−	−	★	★	−	5
Noseworthy PA, 2015	★	★	−	★	★	★	★	★	−	7
Bunch TJ, 2013	★	★	★	★	−	−	★	★	−	6
Lin YJ, 2012	★	★	★	★	★	★	★	★	−	8
Reynolds MR, 2012	★	★	★	★	★	★	★	★	−	8
Chang CH, 2014	★	★	★	★	−	−	★	★	−	6
Friberg L, 2016	★	★	★	★	★	★	−	★	−	7
Jarman JW, 2017	★	★	★	★	★	−	★	★	−	7
Saliba W, 2017	★	★	★	★	★	★	★	★	−	8
Geng J, 2017	★	★	★	★	★	★	★	★	−	8
Srivatsa UN, 2018	★	−	★	★	★	★	★	★	−	7

(1) Indicates the exposed cohort was truly representative; (2) the non-exposed cohort was drawn from the same community; (3) ascertainment of exposure;(4) the outcome of interest was not present at the start of the study; (5A) cohorts were comparable on the basis of the $CHADS_2$ score or $CHA_2DS_2\text{-}VAS_c$ score; (5B) cohorts were comparable in other factor(s); (6) quality of outcome assessment; (7) follow-up was long enough for outcomes to occur (at least 6 months); (8) complete accounting for cohorts(>80% follow-up or description provided of those lost).

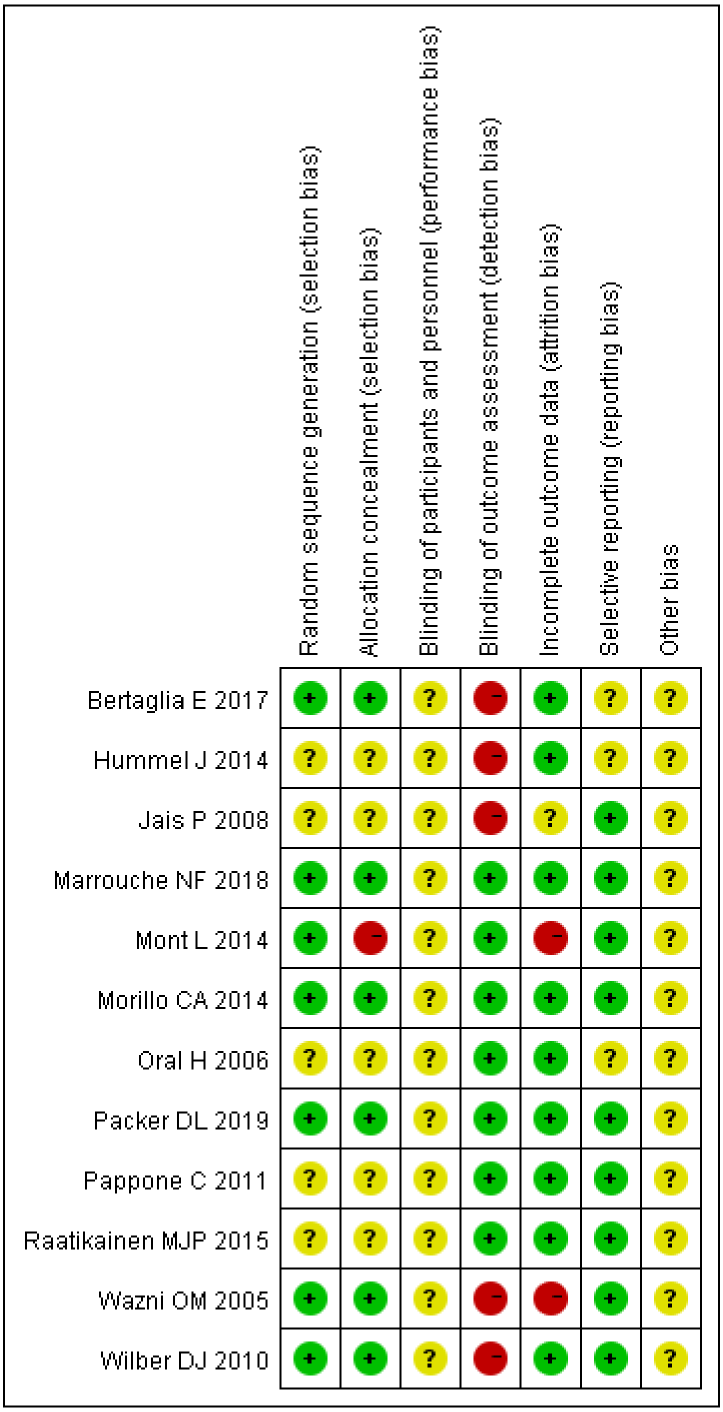

Figure S1. Summary of assessment of the risk of bias in RCTs. Green (+) indicates a low risk of bias; red (-), a high risk of bias; and yellow (?), an unclear risk of bias.

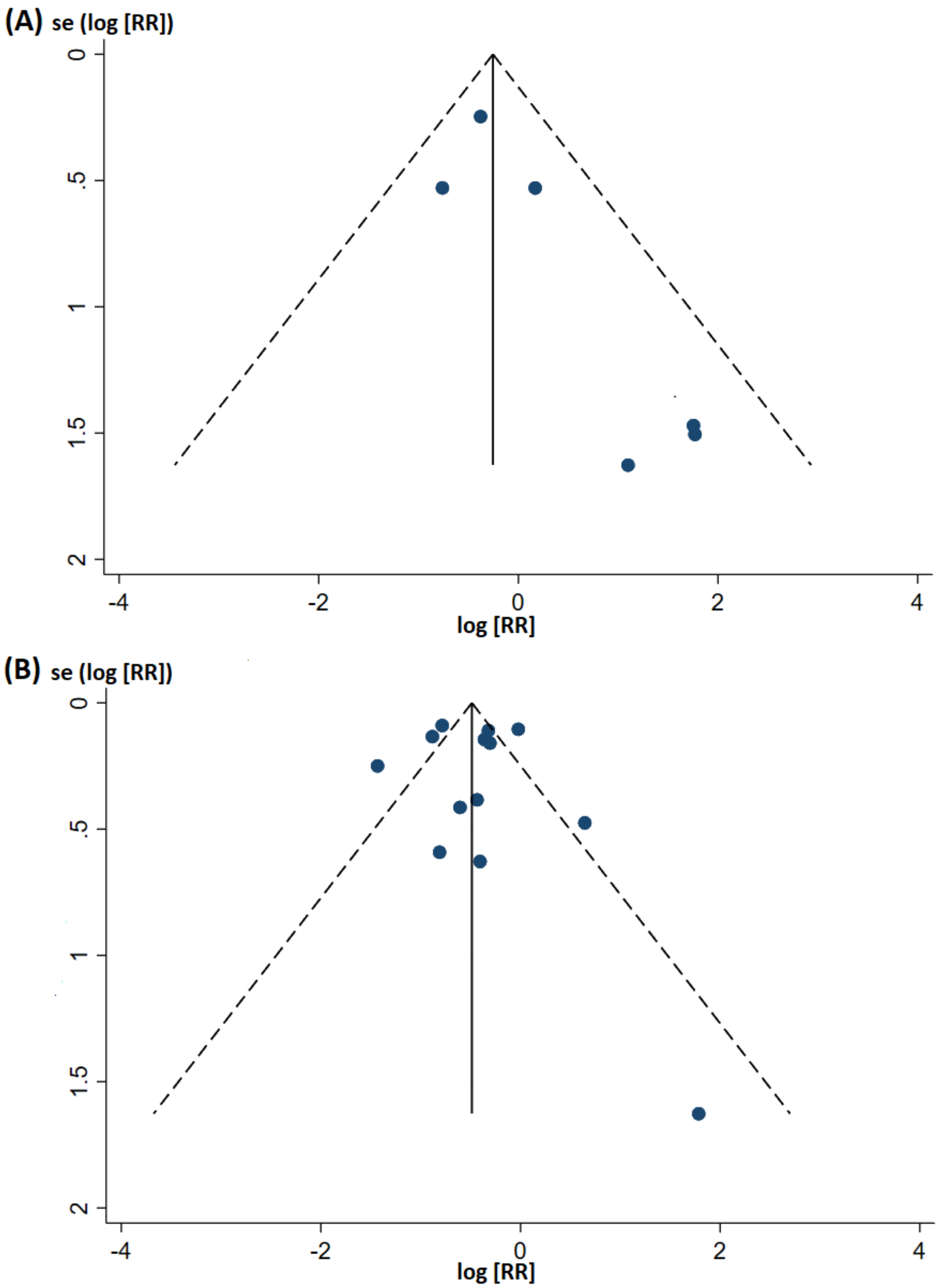

Figure S2. Funnel plot for total thromboembolic events. (A) RCTs; (B) Observational studies.

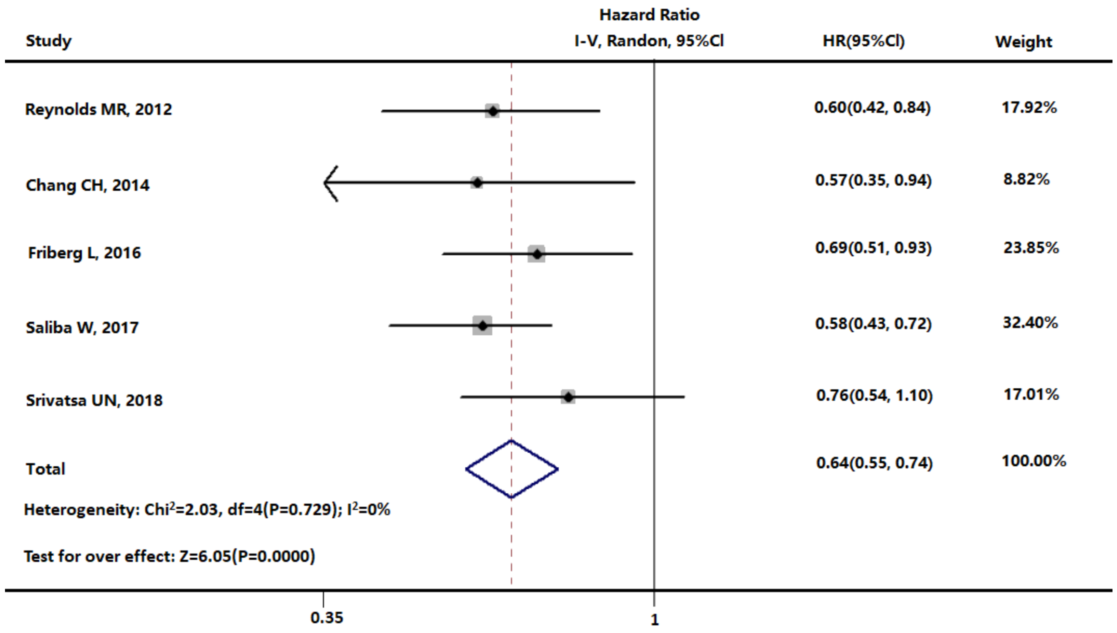

Figure S3. Comparison of the incidence of total thromboembolic events between ablation and non-ablation using multivariable adjusted HR in observational studies.

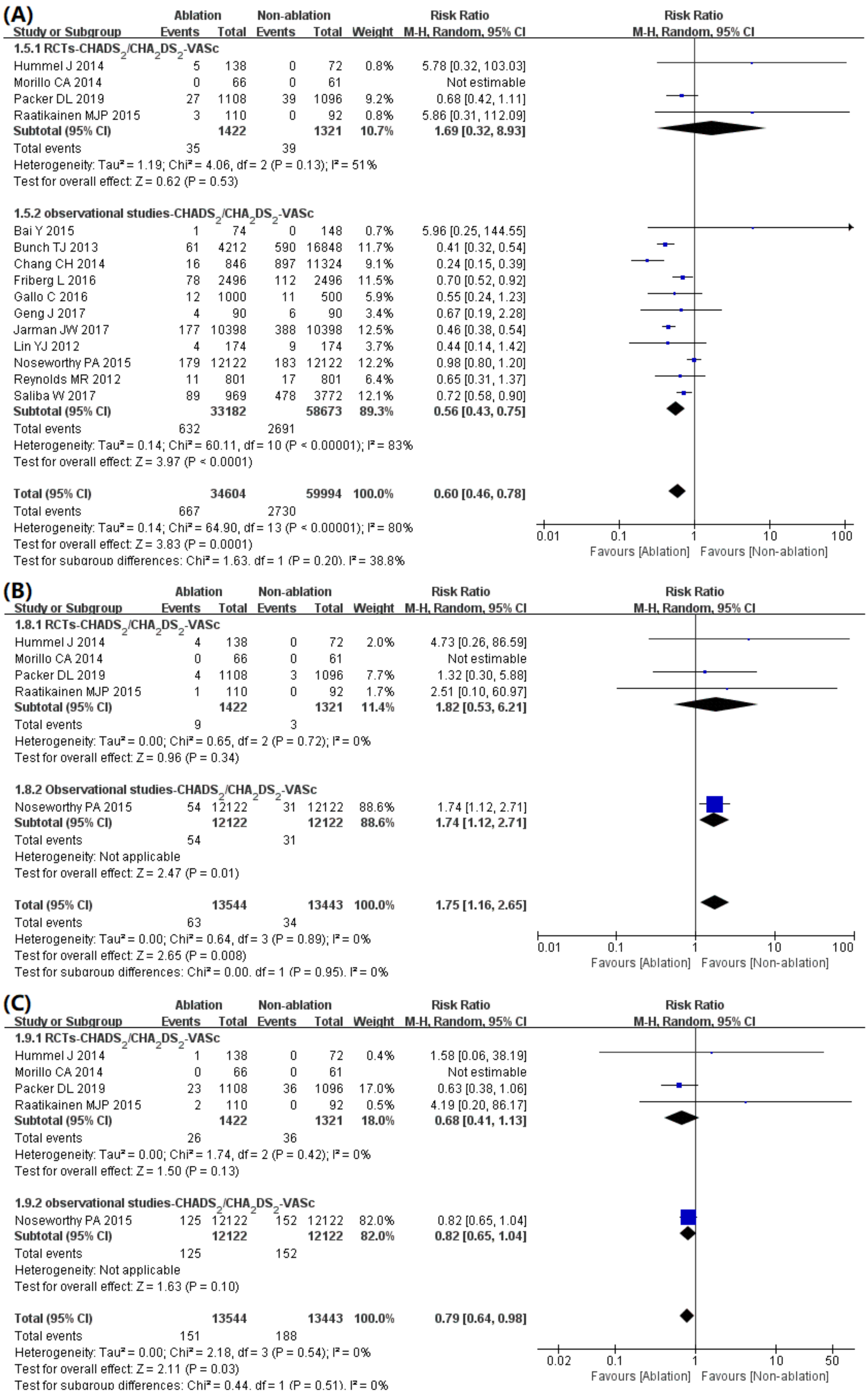

Figure S4. Comparison of the incidence of thromboembolism between ablation and non-ablation after matching the $CHADS_2$/CHA_2DS_2-VASc score. (A) Total thromboembolic events; (B) Early-phase thromboembolic events; (C) Late-phase thromboembolic events.

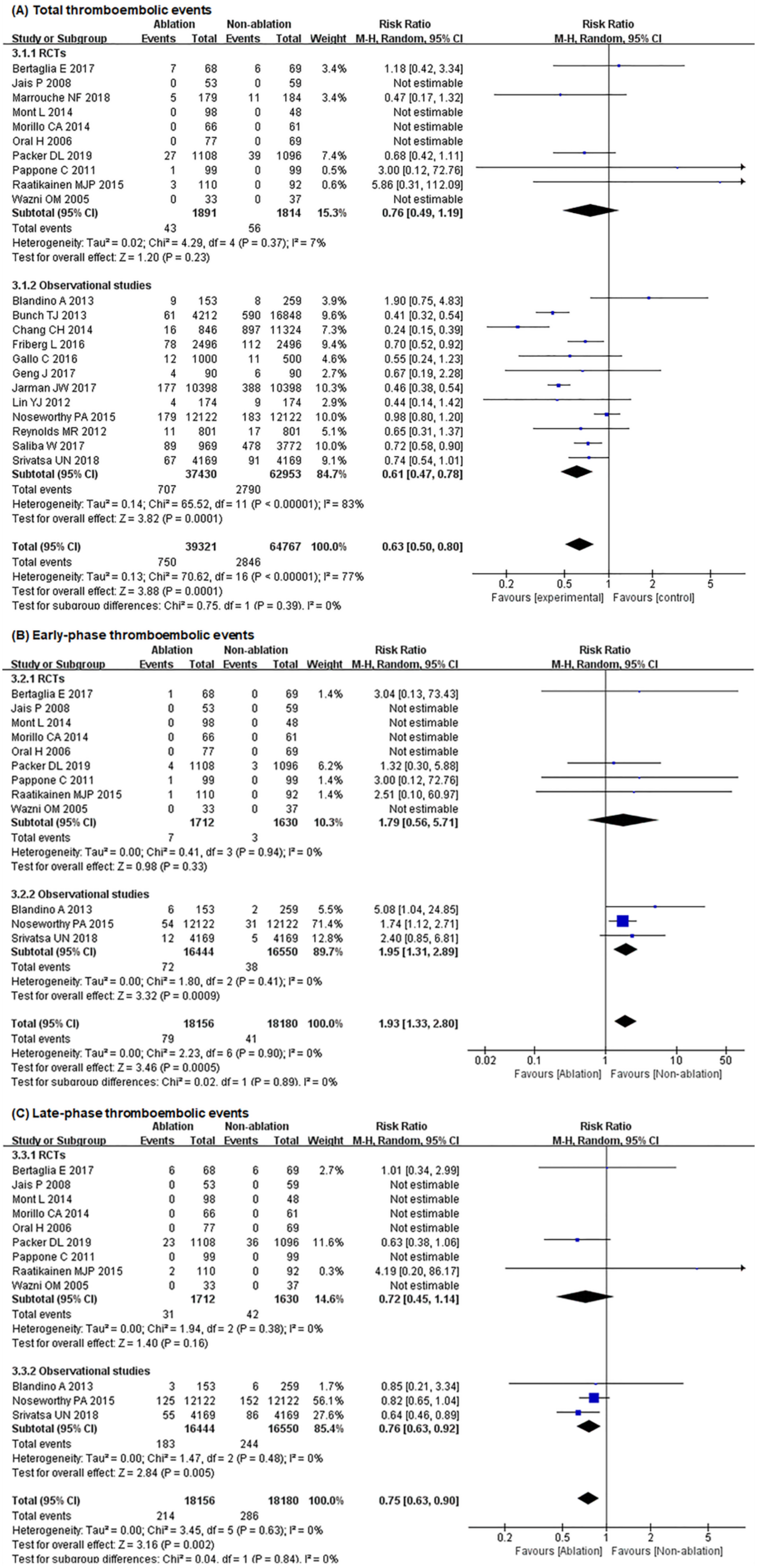

Figure S5. Comparison of the incidence of thromboembolism between ablation and non-ablation in the 22 long-term follow-up studies (follow-up time ≥ 12 months).

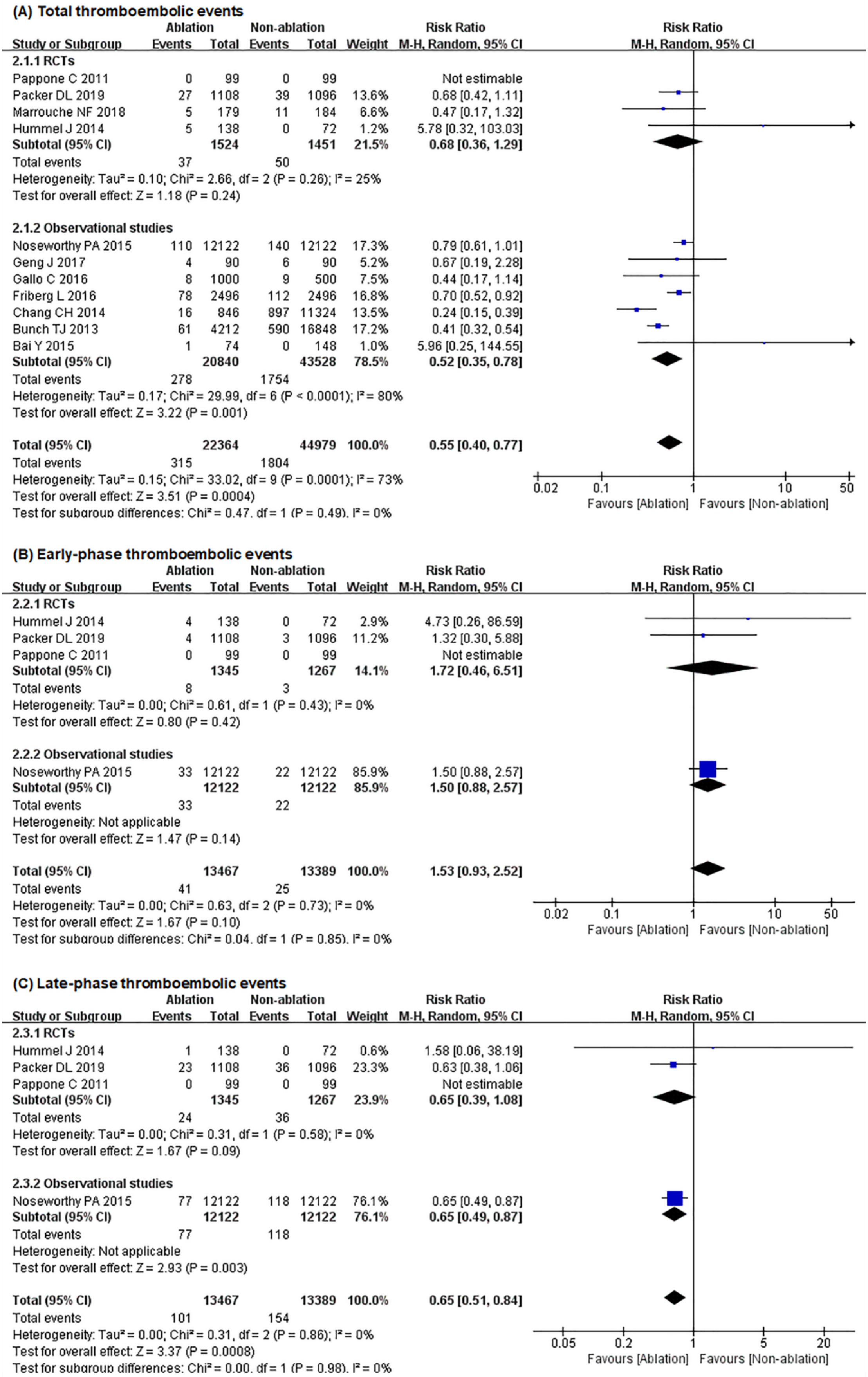

Figure S6. Comparison of the incidence of thromboembolism (only including stroke and systemic embolism) between ablation and non-ablation in 11 studies that have clearly distinguished the different types of thromboembolic events.

Study or Subgroup	Ablation Events	Ablation Total	Non-ablation Events	Non-ablation Total	Weight	Risk Ratio M-H, Random, 95% CI
**1.4.1 RCTs**						
Bertaglia E 2017	7	68	6	69	6.3%	1.18 [0.42, 3.34]
Hummel J 2014	5	138	0	72	1.1%	5.78 [0.32, 103.03]
Jais P 2008	0	53	0	59		Not estimable
Marrouche NF 2018	5	179	11	184	6.3%	0.47 [0.17, 1.32]
Mont L 2014	0	98	0	48		Not estimable
Morillo CA 2014	0	66	0	61		Not estimable
Oral H 2006	0	77	0	69		Not estimable
Packer DL 2019	27	1108	39	1096	14.3%	0.68 [0.42, 1.11]
Pappone C 2011	1	99	0	99	0.9%	3.00 [0.12, 72.76]
Raatikainen MJP 2015	3	110	0	92	1.0%	5.86 [0.31, 112.09]
Wazni OM 2005	0	33	0	37		Not estimable
Wilber DJ 2010	0	106	0	61		Not estimable
**Subtotal (95% CI)**		**2135**		**1947**	**29.8%**	**0.85 [0.49, 1.47]**
Total events	48		56			
Heterogeneity: Tau² = 0.10; Chi² = 6.26, df = 5 (P = 0.28); I² = 20%						
Test for overall effect: Z = 0.57 (P = 0.57)						
**1.4.2 Observational studies**						
Blandino A 2013	9	153	8	259	7.3%	1.90 [0.75, 4.83]
Bunch TJ 2013	61	4212	590	16848	19.2%	0.41 [0.32, 0.54]
Gallo C 2016	12	1000	11	500	8.7%	0.55 [0.24, 1.23]
Lin YJ 2012	4	174	9	174	5.3%	0.44 [0.14, 1.42]
Reynolds MR 2012	11	801	17	801	9.5%	0.65 [0.31, 1.37]
Saliba W 2017	89	969	478	3772	20.1%	0.72 [0.58, 0.90]
**Subtotal (95% CI)**		**7309**		**22354**	**70.2%**	**0.63 [0.43, 0.93]**
Total events	186		1113			
Heterogeneity: Tau² = 0.13; Chi² = 17.27, df = 5 (P = 0.004); I² = 71%						
Test for overall effect: Z = 2.32 (P = 0.02)						
**Total (95% CI)**		**9444**		**24301**	**100.0%**	**0.69 [0.51, 0.94]**
Total events	234		1169			
Heterogeneity: Tau² = 0.11; Chi² = 24.83, df = 11 (P = 0.010); I² = 56%						
Test for overall effect: Z = 2.37 (P = 0.02)						
Test for subgroup differences: Chi² = 0.76, df = 1 (P = 0.38), I² = 0%						

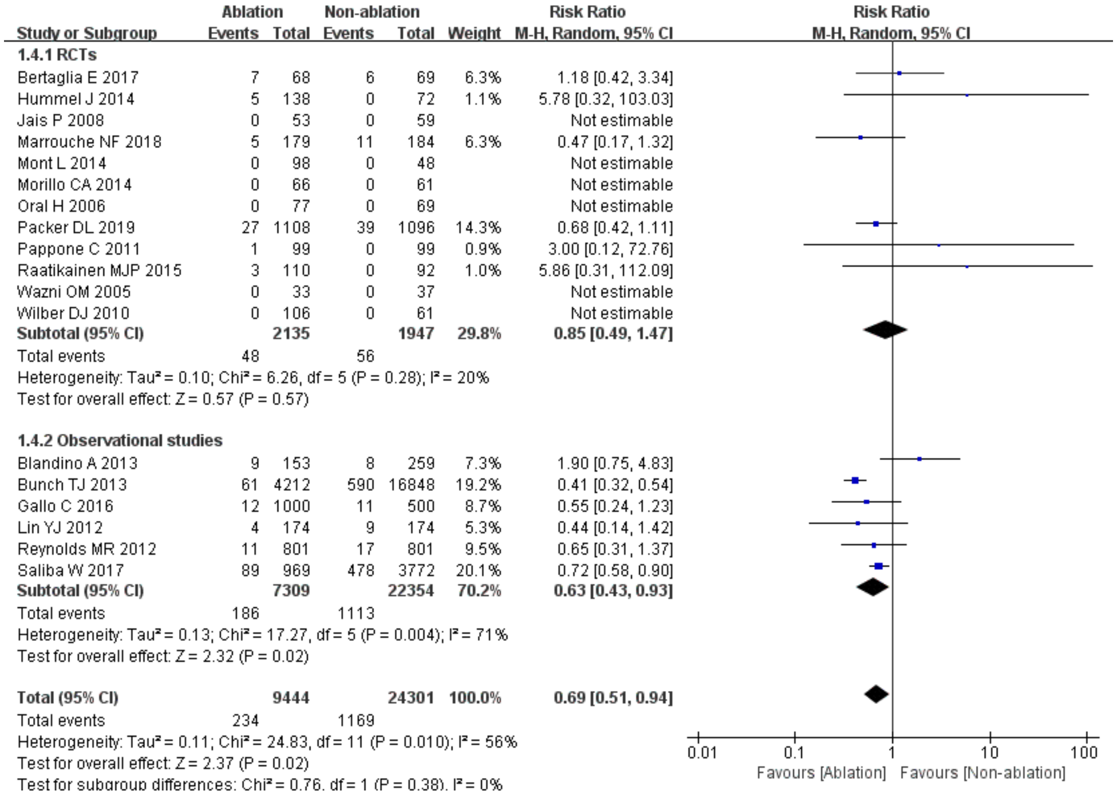

Figure S7. Comparison of the incidence of total thromboembolic events between ablation and non-ablation in studies that described the anticoagulation strategies.
